# Distribution Patterns of Astrocyte Populations in the Human Cortex

**DOI:** 10.1007/s11064-022-03700-2

**Published:** 2022-08-05

**Authors:** Shelley L. Forrest, Jordan Hanxi Kim, Daniel R. Crockford, Katharine Huynh, Rosie Cheong, Samantha Knott, Madison A. Kane, Lars M. Ittner, Glenda M. Halliday, Jillian J. Kril

**Affiliations:** 1 Dementia Research Centre, School of Biomedical Sciences, Faculty of Medicine, Health and Human Sciences, Macquarie University, 13A Research Park Drive, Sydney, NSW 2109 Australia; 2 Faculty of Medicine and Health, School of Medical Sciences, University of Sydney, Sydney, Australia; 3 Brain and Mind Centre, Faculty of Medicine and Health, University of Sydney, Sydney, Australia

**Keywords:** Astrocyte, Human cortex, Aquaporin-4, Connexin 43, Glutamate transporter 1, Glial fibrillary acidic protein

## Abstract

Astrocytes are a major class of glial cell in the central nervous system that have a diverse range of types and functions thought to be based on their anatomical location, morphology and cellular properties. Recent studies highlight that astrocyte dysfunction contributes to the pathogenesis of neurological conditions. However, few studies have described the pattern, distribution and density of astrocytes in the adult human cortex. This study mapped the distribution and density of astrocytes immunolabelled with a range of cytoskeletal and membrane markers in the human frontal cortex. Distinct and overlapping astrocyte populations were determined. The frontal cortex from ten normal control cases (75 ± 9 years) was immunostained with glial fibrillary acidic protein (GFAP), aldehyde dehydrogenase-1 L1 (ALDH1L1), connexin-43 (Cx43), aquaporin-4 (AQP4), and glutamate transporter 1 (GLT-1). All markers labelled populations of astrocytes in the grey and white matter, separate cortical layers, subpial and perivascular regions. All markers were informative for labelling different cellular properties and cellular compartments of astrocytes. ALDH1L1 labelled the largest population of astrocytes, and Cx43-immunopositive astrocytes were found in all cortical layers. AQP4 and GLT-1 labelled distal astrocytic process and end-feet in the same population of astrocytes (98% of GLT-1-immunopositive astrocytes contained AQP4). In contrast, GFAP, the most widely used marker, predominantly labelled astrocytes in superficial cortical layers. This study highlights the diversity of astrocytes in the human cortex, providing a reference map of the distribution of distinct and overlapping astrocyte populations which can be used for comparative purposes in various disease, inflammatory and injury states involving astrocytes.

## Introduction

The human brain is comprised of a complex array of cell types including glia, which are a diverse population of non-neuronal cells. Glia form complex networks and interactions with other glial cells and neurons, and it is now well-established that glia have a wide range of critical functions beyond structural support for neurons. Astrocytes are a major class of glial cell that have been receiving increased attention in studies of central nervous system physiology and dysfunction, and in the context of neurodegenerative disorders [1–4]. Astrocytes provide homeostatic control at all levels of organisation in the central nervous system including the regulation of ionic and neurotransmitter homeostasis, providing energy substrates to surrounding neurons, modulating synaptic transmission, secretion of trophic factors, maintenance of the blood-brain-barrier, and reacting to local insults and inflammation [2, 5]. Furthermore, there is evidence of the important role of astrocytes in the pathogenesis of a range of neurological conditions [6–8], including neurodegenerative diseases, and their involvement in brain ageing [9–12].

A number of astrocyte types are recognised, which are classified based on their anatomical location, morphology, function, and cellular properties [2, 13]. Protoplasmic astrocytes, located in the grey matter, are characterised by a small soma with elaborate processes, which are classified into branches and branchlets, leaflets and endfeet that occupy distinct territorial domains. Fibrous astrocytes, located in the white matter, are characterised by a smaller diameter cell body, and straight, non-branched processes [2, 5, 13, 14]. The distal processes of protoplasmic and fibrous astrocytes also make connections with blood vessels and create perivascular end-feet [2, 4]. The cytoskeleton of astrocytes is dominated by intermediate filaments and comprise microtubules and actin filaments. Glial fibrillary acidic protein (GFAP) is a major constituent of astrocyte intermediate filaments, and antibodies to this protein are the most commonly used immunohistochemical marker for the detection of astrocytes in rodent and human tissue [1, 2, 15]. However, while GFAP and other cytoskeletal markers of astrocytes, including aldehyde dehydrogenase-1 family, member L1 (ALDH1L1), allow visualisation of the cell body and major proximal processes, the finer astrocytic processes are not detected. Astrocytes express several ion channels, water channels (aquaporins), connexins, neurotransmitters and neuro-transporters. However, these markers frequently produce widespread and diffuse immunostaining, and the identification of complex astrocytic morphologies remains challenging. Gap junctions and hemichannels are composed of connexins, which maintain the normal morphology and function of astrocytes, and allow transfer of molecules between cells and the extracellular matrix. Connexin-43 (Cx43) is highly expressed by astrocytes, particularly at the blood-brain-barrier, and is recognised as a functional entity capable of influencing metabolic gradients [16, 17]. Aquaporin-4 (AQP4) is the primary water channel protein expressed by astrocytic end-feet surrounding capillaries, and is associated with water transfer into and out of the brain parenchyma [18]. The glutamate transporter 1 (GLT-1)/excitatory amino acid transporter 2, is the principal transporter responsible for clearing glutamate from neuronal synapses and the extracellular space, and is specifically expressed by astrocytes [19]. The complex morphological heterogeneity of astrocytes correlates with the diversity in expression of these markers, which identify different sub-populations of astrocytes with regional anatomical variation.

While a wide range of protein markers are used to label astrocytes and identify their specific functions and processes [20], the majority are used in in vivo experiments and rodent tissue, and few are reliably used in human postmortem tissue. Few studies have described the distribution and diversity of astrocytes in human tissue in detail. In addition, human astrocytes are larger, with far greater diversity and complexity than astrocytes in rodents, and various staining techniques, for example, Golgi and GFAP immunostaining, can lead to misinterpretation of morphology and classes of astrocytes [2, 21]. This study mapped the distribution and density of astrocytes immunolabelled with a range of cytoskeletal and membrane markers in the human frontal cortex. In addition, distinct and overlapping astrocytic populations were identified.

## Materials and Methods

### Case Selection

Ten control cases (5 male; mean age: 75 ± 9 years; range: 60–87 years) were selected for this study from the Sydney Brain Bank. Participants were prospectively enrolled in longitudinally multidisciplinary research programs and recruited with informed consent through regional brain donor programs. Sydney Brain Bank holds ethics approval from the University of New South Wales (Sydney). Research conducted in this study was approved by the University of Sydney and Macquarie University Human Research Ethics Committees and complies with the statement on human experimentation issued by the National Health and Medical Research Council of Australia.

Demographic information was collected from an integrated clinicopathological database. All cases included in this study had a routine neuropathological assessment using standardised neuropathological consensus recommendations. Cases with co-existing pathologies, including high level Alzheimer’s disease (AD) neuropathological change and Lewy body disease [22, 23], or significant vascular pathology were excluded [24, 25]. Four cases had low level AD neuropathological change, one case had intermediate level AD neuropathological change, and five cases had no AD neuropathological change [24]. There was little or no cerebral amyloid angiopathy (CAA) or arteriolosclerosis in the cases examined, and none in the brain region examined (superior frontal cortex).

### Assessment of Astrocytes

Formalin-fixed paraffin-embedded 10 μm sections from the superior frontal cortex in all cases were immunostained with glial fibrillary acidic protein (GFAP; Agilent; Cat. No. Z033401; rabbit; 1:2000), ALDH1L1 (UltraLab; Cat. No. UM570040; mouse; 1:1000), Cx43 (Abcam; Cat. No. ab11370; rabbit; 1:500), AQP4 (Sigma; Cat. No. A5971; rabbit; 1:2000), and glutamate transporter 1/ excitatory amino acid transporter 2 (GLT-1; Merck/Millipore; Cat. No. AB1783; guinea pig; 1:2000). All immunoperoxidase-stained sections were counterstained with haematoxylin. Three cases were selected for double-labelled immunofluorescence using a combination of astrocytic markers to determine overlapping populations of astrocytes. All immunofluorescent sections were counterstained with 4′,6-diamidino-2-phenylindole (DAPI) to identify nuclei.

All immunostained sections were semi-qualitatively analysed and the distribution and density of astrocytes immunolabelled with each marker were based on the following criteria: (1) presence of well-defined astrocytes, with clearly distinguishable cell body and/or processes within individual domains; (2) location of immunoreactivity in the cell body, proximal or distal processes; (3) presence/distribution of astrocytes in cortical layers I/II, III/IV, V/VI, white matter (gyral and/or deep), perivascular regions (grey and white matter); (4) density of immunolabelled astrocytes in each area as none (0), sparse (1), moderate (2), high (3). The density of astrocytes immunolabelled with each marker is reported as the percentage of cases with none, sparse, moderate, or high density of astrocytes in each specified location. Double-labelled counts were performed on three cases and were obtained from the middle cortical layers where discrete astrocytic domains were observed. Values and are reported as the mean percentage.

Immunoperoxidase sections were viewed under an Olympus BX51 microscope and counts of double-labelled astrocytes were also performed on a BX51 microscope. Images were captured with a Leica SPII confocal microscope under an ACS APO 40 × oil-immersion objective with a Leica DFC camera. Lasers of excitatory wavelengths 405 nm, 488 nm, and 532 nm were used to excite fluorophores. Only minor adjustments to brightness and contrast were made to images with Adobe Photoshop CC to best capture immunostaining as viewed directly under the microscope.

## Results

### Distribution Patterns and Density of Astrocytes Labelled with Each Marker

#### Glial Fibrillary Acidic Protein (GFAP)

**Fig. 1:**
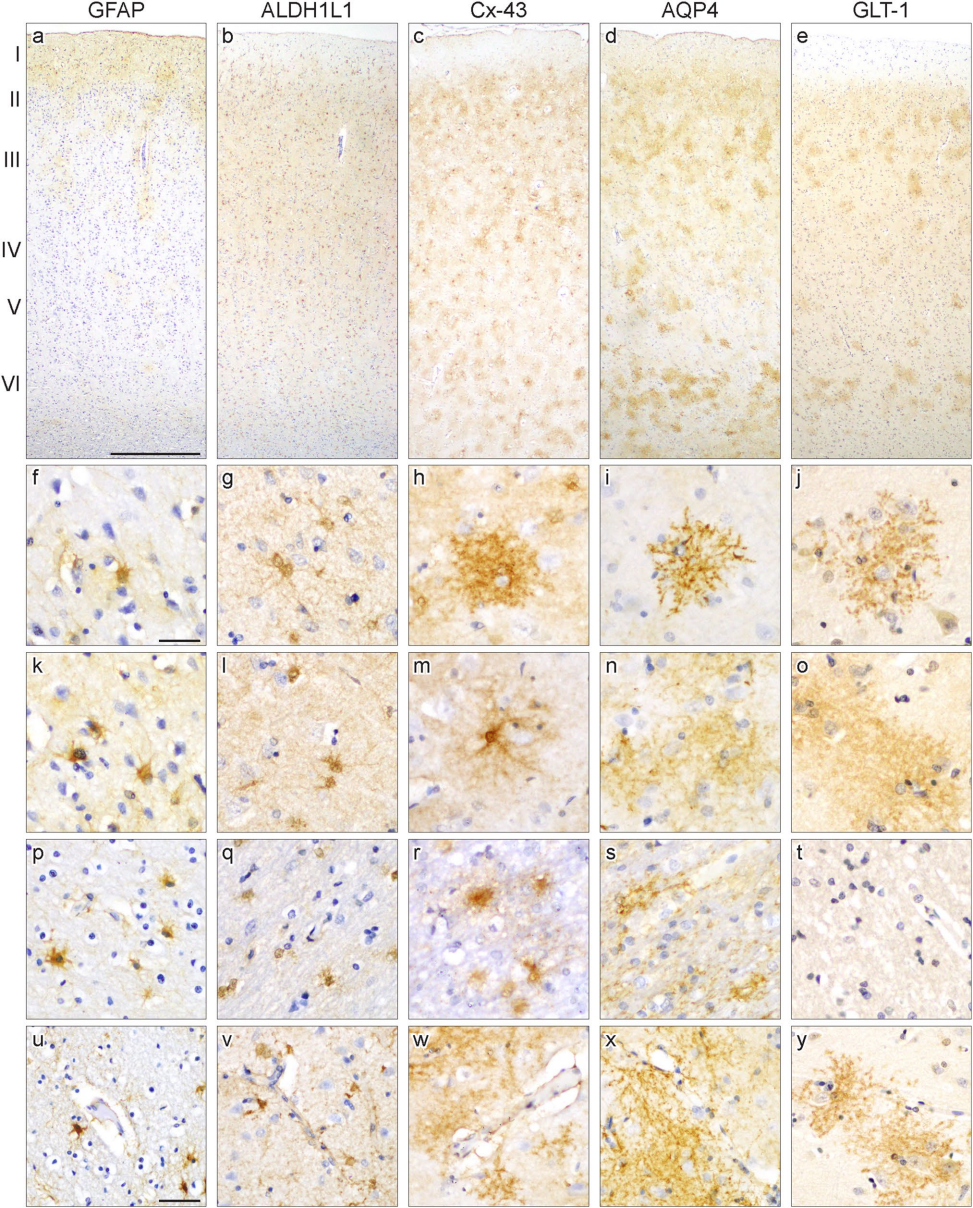
Representative images of astrocytes immunolabelled with each antibody in the superior frontal cortex. The images in each column are taken from the same case and all sections are counterstained with hematoxylin. The pial surface is orientated to the top in panels a-e with the cortical layers depicted. Immunostaining with glial fibrillary acidic protein (GFAP; (**a**, **f**, **k**, **p**, **u**)) and aldehyde dehydrogenase-1 L1 (ALDH1L1; (**b**, **g**, **l**, **q**, **v**)) label the astrocytic cell body and processes. ALDH1L1 labelled the largest proportion of astrocytes, which were distributed throughout all cortical layers. Connexin-43 (Cx43; (**c**, **h**, **m**, **r**, **w**)), aquaporin-4 (AQP4; (**d**, **i**, **n**, **s**, **x**)) and glutamate transporter-1 (GLT-1; (**e**, **j**, **o**, **t**, **y**)) label astrocytic processes with intense or diffuse immunoreactivity observed. Discrete astrocytic territories could be observed with these markers (**h**–**j**). Occasional Cx43-immunoreactivity was observed in the cell body of astrocytes (**m**). Astrocytes and/or their processes immunolabelled with GFAP, ALDH1L1, Cx43 and AQP4 were observed in the frontal white matter (**p**–**s**). GLT-1-immunopositive astrocytes or processes were not observed in the white matter (**t**). Astrocytes immunolabelled with each marker were observed close to blood vessels in the grey and white matter (**u**–**y**). Scale bar in a = 500 μm (applies **b**–**e**); 20 μm in **f** (applies **g**–**t**); 50 μm in **u** (applies **v**–**y**)

**Fig. 2:**
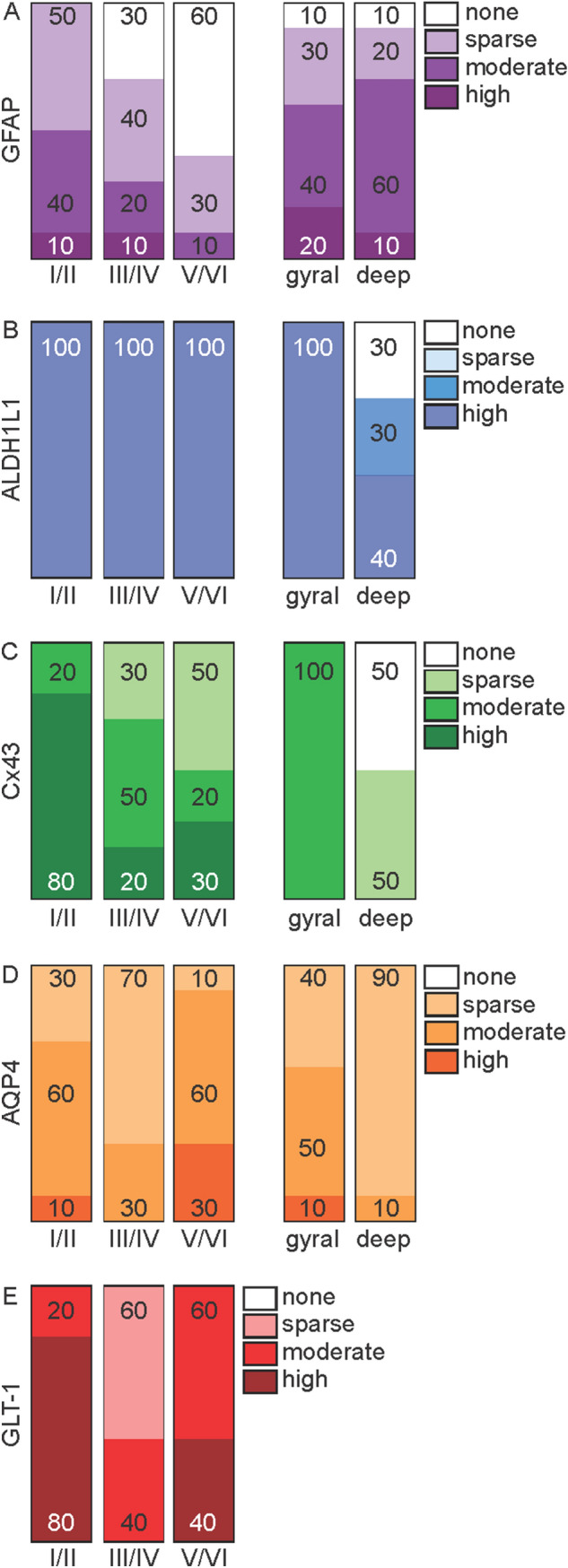
Bar graphs showing the anatomical distribution and density of astrocytes expressing each marker in the grey and white matter. Density is reported as % of cases with none, sparse, moderate or high density of astrocytes immunolabelled with GFAP (**a**), ALDH1L1 (**b**), Cx43 (**c**), AQP4 (**d**) and GLT-1 (**e**) in grey (layers I/II, III/IV, V/VI) and white (gyral, deep) matter

GFAP-immunostaining was observed in the cell body of astrocytes with intense immunoreactivity in the cell body and proximal astrocytic processes in all cases (Fig. 1a, f, k, p). Fine astrocytic processes and end-feet were not labelled. GFAP-immunopositive astrocytes were observed individually or in small clusters in the grey and white matter. GFAP-immunopositive astrocytes were generally distributed throughout all cortical layers, and the distribution and density of astrocytes immunostained with GFAP varied between cases. GFAP-immunopositive astrocytes were observed in layers I/II in all cases, in layers III/IV in seven cases, and in layers V/VI in four cases (Table 1, Fig. 2a). The highest density of GFAP-immunopositive astrocytes was observed in cortical layers I–II, followed by layers III/IV, and layers V/VI. GFAP-immunopositive astrocytes were observed in both the gyral and deep frontal white matter, and a cluster at the grey-white junction was found in half of all cases. Occasional GFAP-immunopositive astrocytes were observed closely associated with blood vessels in grey and white matter without obvious vascular pathology (Fig. 1u).

**Table 1:** Anatomical distribution and percentage of cases expressing each astrocytic marker in the cytoplasm and processes

	GFAP	ALDH1L1	Cx43	AQP4	GLT-1
Grey matter					
Well-defined astrocytes	100	100	80	70	100
Cell body	100	100	80	0	0
Processes	100	80	100	100	100
Perivascular	100	100	80	100	100
Layers (% cases/density)*					
I/II	100 (0,50,40,10)	100 (0,0,0,100)	100 (0,0,20,80)	100 (0,30,60,10)	100 (0,0,20,80)
III/IV	70 (30,40,20,10)	100 (0,0,0,100)	100 (0,30,50,20)	100 (0,70,30,0)	100 (0,60,40,0)
V/VI	40 (60,30,10,0)	100 (0,0,0,100)	100 (0,50,20,30)	100 (0,10,60,30)	100 (0,0,60,40)
Layers I/II > other (% cases)	100	0	50	90	60
Layers III/IV < other (% cases)	0	0	30	10	0
White matter					
Well-defined astrocytes	100	100	80	100	0
Cell body	100	100	100	0	0
Processes	100	80	80	100	0
Perivascular	80	80	100	90	0
Gyral white matter (% cases/density)*	90 (10,30,40,20)	100 (0,0,0,100)	100 (0,0,100,0)	100 (0,40,50,10)	0
Deep white matter (% cases/density)*	90 (10,20,60,10)	70 (30,30,0,40)	50 (50,50,0,0)	100 (0,90,10,0)	0
Gyral > deep	10	100	100	100	0

^*^Density reported as % of cases with none (0), sparse (1), moderate (2), or high (3)

#### Aldehyde Dehydrogenase-1 Family, Member L1 (ALDH1L1)

ALDH1L1 clearly labelled the cell body, and proximal and distal processes in the frontal grey and while matter (Fig. 1b, g, l, q). Occasional fine astrocytic processes were observed with ALDH1L1. ALDH1L1-immunopositive astrocytes were observed individually or in small clusters, and all astrocytes were well-defined. In all cases, ALDH1L1-immunopositive astrocytes were evenly distributed throughout all cortical layers with a similar high density throughout all layers (Table 1, Fig. 2b). In the white matter, ALDH1L1-immunopositive astrocytes were observed in both the gyral and deep white matter. Half of all cases showed a clustering of ALDH1L1-immunopostive astrocytes at the grey-white junction. Many ALDH1L1-immunopositive astrocytes were observed in close proximity to blood vessels in grey and white matter with astrocytic processes extending close to, or ending, on blood vessels (Fig. 1v). The pattern and density of astrocytes immunolabelled with ALDH1L1 was similar between all cases.

#### Connexin-43 (Cx43)

Immunostaining with Cx43 revealed staining in the astrocytic cell body and processes (Fig. 1c). Cx43-immunostaining varied between astrocytes with either intense immunoreactivity or diffuse puncta in the cell body. Proximal and distal astrocytic processes contained either Cx43-immunopositive fine dots or fine and diffuse staining (Fig. 1h, m). 9 out of 10 control cases had well-defined astrocytes labelled with Cx43, with discrete astrocytic territories frequently observed. Cx43-immunopositive astrocytes were observed individually or in dense clusters of astrocytes where individual astrocyte territories could not be distinguished. Occasional astrocytic processes were observed making contact with neighbouring Cx43-immunopositive astrocytes. In all cases, Cx43-immunopositive astrocytes were distributed throughout all cortical layers, and the density of astrocytes varied between layers and cases (Table 1, Fig. 2c). Cx43-immunopositive astrocytes were frequently observed in the frontal gyral white matter (Fig. 1r), and were less commonly observed in the deep white matter. The cell body and processes of astrocytes in the white matter immunolabelled with Cx43 appeared more diffuse than those observed in the grey matter. Frequent Cx43-immunopositive astrocytes were observed close to blood vessels in grey and white matter, with astrocytic processes connecting to the vessel wall (Fig. 1w).

#### Aquaporin 4 (AQP4)

AQP4 predominantly labelled astrocytic processes in the frontal grey and white matter, and occasional immunostaining was observed in the cell body (Fig. 1d). Diffuse and punctate AQP4-immunostaining was observed in proximal and distal astrocytic processes, which extended to the end-feet (Fig. 1i, n). Like Cx43-immunostaining, astrocytes labelled with AQP4 were generally well defined, with discrete astrocytic territories observed in 7 out of 10 cases. The remaining three cases had poorly defined astrocytes, characterised by diffuse immunostaining. AQP4-immunopositive astrocytes were observed in isolation or large clusters of astrocytes, where individual territories could not be distinguished. Astrocytes were distributed throughout all cortical layers with the highest density in layers I/II and V/VI. Cortical layers III/VI had a lower density of AQP4-immunopositive astrocytes than superficial and deep layers (Table 1, Fig. 2d). A prominent feature of AQP4-immunostaining was the preferential distribution of astrocytes to perivascular regions with numerous astrocytes observed close to blood vessels (Fig. 1x). Their distal processes and end-feet frequently made contact with capillaries and small-diameter vessels in the frontal grey matter. Numerous astrocytes were also observed close to white matter vessels. AQP4-immunopositive astrocytes were found in the gyral and deep white matter, with a higher density in the gyral white matter (Fig. 1s), and a cluster at the grey-white junction was observed in most cases.

#### Glutamate Transporter 1 (GLT-1)

Immunostaining with GLT-1 revealed a similar pattern and distribution of astrocytes as observed with AQP4. GLT-1 clearly labelled the astrocytic membrane with large, punctate immunostaining observed in astrocytic distal processes and end-feet (Fig. 1e). Immunostaining in the cell body was not observed with GLT-1 (Fig. 1j, o). GLT-1-immunopositive astrocytes were predominantly located in layers I/II and V/VI and the highest density of astrocytes were observed here. Middle cortical layers III/IV contained a lower density of GLT-1-imunopositive astrocytes (Table 1, Fig. 2e). Similar to AQP4, numerous GLT-1-immunopositive astrocytes were associated with perivascular regions, with their end-feet frequently making contact with capillaries and small-diameter vessels (Fig. 1y). Unlike other astrocytic markers, GLT-1-immunopositive astrocytes, including their processes, were not observed in the white matter (Fig. 1t).

#### Overlapping and Distinct Astrocytic Populations

**Fig. 3:**
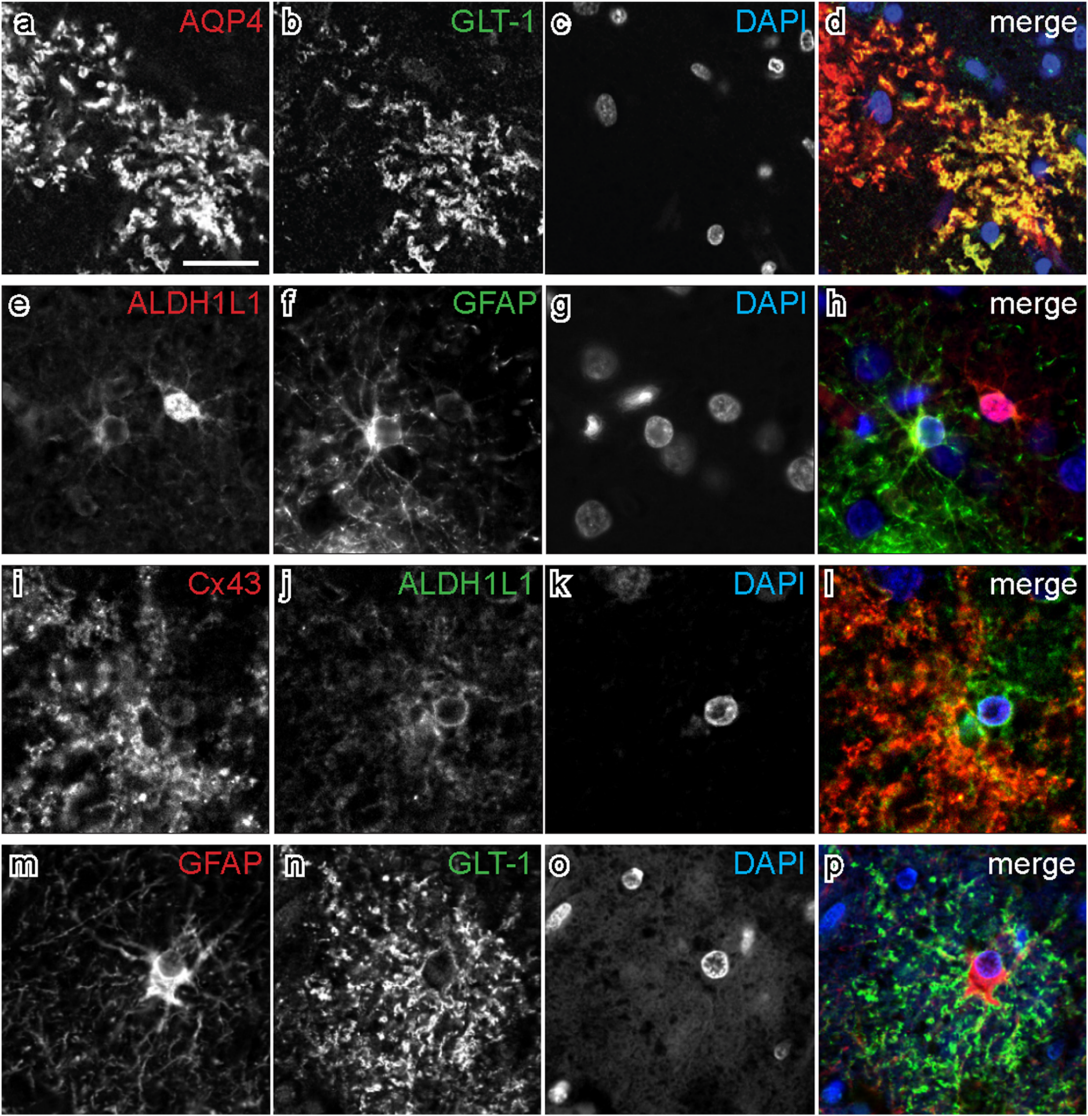
Double-labelled immunofluorescence shows co-expression of different astrocyte markers. Each horizontal set comprises images taken from the same field of view, showing immunostaining for different markers in the superior frontal cortex. The colour of each label in the merged image is represented by the colour of the text in the relevant monochrome image. Sections were counterstained with DAPI to identify nuclei. **a–d.** Aquaporin-4 (AQP4) and glutamate transporter 1 (GLT-1) both labelled astrocytic processes and end feet, and are a largely overlapping population of astrocytes. **e–h**. Aldehyde dehydrogenase-1 L1 (ALDH1L1) and glial fibrillary acidic protein (GFAP) label the cell body and processes of astrocytes. **i–l**. An astrocyte immunolabelled with connexin-43 (Cx43) and ALDH1L1. **m–p**. Different cellular compartments are labelled with GFAP and GLT-1, and are largely a non-overlapping population of astrocytes. Scale bar in a = 20 μm (applies to all images)

Immunofluorescent double-labelling with select astrocytic markers was used to determine overlapping and distinct astrocytic populations identified by single-labelling. Almost all GLT-1-imunopositive astrocytes contained AQP4-immunoreactivity (98%), and 79% of AQP4-immunopositive astrocytes contained GLT-1-immunoreactivity, which confirmed the observations above that these two markers are mostly labelling the same population of astrocytes. Confocal microscopy also revealed that immunoreactivity for both makers was observed in the same cellular compartments of distal astrocytic processes and end-feet (Fig. 3a–d). Observations with single-labelled ALDH1L1 immunostaining revealed that this antibody identified the largest population of astrocytes, which was confirmed with double-labelled immunofluorescence. Almost all (99%) GFAP-immunopositive astrocytes contained ALDH1L1-immunoreactivity, of which 87% contained GFAP-immunoreactivity (Fig. 3e–h). Similar cellular compartments were labelled with both antibodies. 35% of ALDH1L1-immunopositive astrocytes contained Cx43 (Fig. 3i–l) and 6% contained GLT-1. Few GLT-1-immunopositive astrocytes were labelled with cytoskeletal markers with 14% and 18% containing ALDH1L1- and GFAP-immunoreactivity, respectively. The majority (79%) of Cx43-immunopositive astrocytes contained ALDH1L1. Similarly, only 14% of GFAP-immunopositive astrocytes contained GLT-1-immunoreactivity, and were largely distinct astrocytic populations that labelled different cellular compartments (Fig. 3m–p). 65% of GLT-1-immunopositive astrocytes contained Cx43, and 87% of Cx43-immunopositive astrocytes contained GLT-1.

## Discussion

This study mapped the distribution of astrocytes immunolabelled with different cytoskeletal and membrane markers. Previously, few studies have described the pattern, distribution, and density of astrocytes in the adult human cortex. All astrocyte markers selected for the current study labelled populations of astrocytes in the grey and white matter, separate cortical layers, and subpial and perivascular regions. In addition, all markers were informative for labelling different cellular properties and cellular compartments of astrocytes. This study further highlights the diversity of astrocytes in the human cortex and provides a reference map of the pattern and distribution of different astrocyte populations, which can be used for comparison to different anatomical locations in the human brain and for comparative purposes in various disease, inflammatory and injury states.

GFAP, the major constituent of intermediate filaments, is the most widely used immunohistochemical marker for the identification of astrocytes, however, many astrocytes do not express GFAP in levels that can be detected with immunohistochemistry [26]. In the normal neocortex, many astrocytes in superficial and deep layers express GFAP, whereas few astrocytes in middle cortical layers express GFAP [26], and similar observations were made in the current study. Upregulation of GFAP protein and mRNA is a feature of reactive astrocytes that undergo morphological, molecular and functional changes in their response to injury and in response to a variety to neuroinflammatory and neurodegenerative disease [1]. For these reasons, a widespread distribution of GFAP-immunopositive astrocytes in the cortex was not expected in the current cohort of control cases. Together, these studies emphasise that combining GFAP with other ubiquitous astrocytic cytoskeletal markers, including ALDH1L1, which labels both GFAP-positive and negative astrocytes [5], is likely to provide a better representation of global astrocyte distribution and density. This study also shows that astrocytes containing AQP4 and GLT-1 are predominantly distributed in superficial and deep cortical layers, and in comparison, astrocytes containing Cx43 have a widespread distribution throughout cortical layers. Recent studies using single-cell transcriptomics have identified the presence of astrocyte layers in the human cortex, similar to the six-layered neocortical structure [27]. These studies show that subsets of astrocytes in the cortical grey matter layers II-VI show heterogeneity in laminar spatial gene expression, suggesting a complex ‘neuroglial’ cortical architecture [27] not previously appreciated, which is likely to have important local and regional functional implications.

Glutamate transporters, namely GLT-1, are responsible for almost all glutamate uptake in the brain and there are notable differences between glutamate metabolism in the grey and white matter [28]. In grey matter, glutamate is released at neuronal synapses to the postsynaptic cell. Glutamate is taken up by astrocytic processes at the synaptic cleft for recycling. In white matter, glutamate is released from axons in smaller quantities than is released from synapses in the grey matter. Consequently, protoplasmic astrocytes in grey matter are exposed to high levels of glutamate and glutamate clearance is high. In contrast, fibrous astrocytes in the white matter are exposed to low levels of glutamate and glutamate clearance is low [29, 30]. Several in vivo and animal studies have reported low expression of glutamate transporters in white matter [21, 28]. Consistent with these studies, the current study did not find GLT-1-expressing astrocytes or their processes in the white matter, which is likely to reflect differences in glutamate metabolism in grey and white matter cortical regions.

Astrocytes are highly metabolically active cells and alterations in their homeostasis and dysfunction are a key feature of many neurodegenerative, neurodevelopmental, psychiatric, inflammatory, prion and other diseases [5, 12, 31–36], and more recently, brain ageing [37]. Recent single-cell transcriptomics studies have identified further complexity in astrocyte function and their response to injury and disease [38, 39], and there is evidence to suggest that astrocytes contribute to the spread of abnormal protein aggregates throughout the brain [6, 9, 12]. The contribution of astrocytes in the pathogenesis of these diseases is largely mediated by alterations in glutamate and ion homeostasis, disruption of energy metabolism, and neurotoxicity, which have been recently reviewed [31]. Cx43, AQP4 and GLT-1 have been examined in the context of many of these diseases and are summarised below.

Connexins are main constituents of gap junctions and different isoforms are recognised. Cx43 is the primary channel protein expressed by astrocytes and the function of Cx43 has been linked to a number of processes that impact brain homeostasis and repair following injury, and neurodegenerative diseases [40]. Cx43 has been shown to perform immunoregulatory roles at the gliovascular interface (blood brain barrier), distribute glucose through the perivascular astroglial network and release gliotransmitters [41]. Increased Cx43-immunostaining in cortical regions containing β-amyloid plaques has been reported in Alzheimer’s disease (AD) [42] and increased Cx43 density has been reported in the caudate nucleus in Huntington’s disease (HD) [43]. Altered Cx43 expression has also been reported in Parkinson’s disease (PD), amyotrophic lateral sclerosis and demyelinating disorders [5, 40, 44]. A recent study in cases with ageing-related tau astrogliopathy (ARTAG) found an increase in Cx43 density that correlated with the density of tau pathology [37]. Whether elevated Cx43 levels in astrocytes are a protective response to maintain tissue homeostasis or further disrupt homeostasis and exacerbate pathological conditions remains to be investigated.

The current study shows that AQP4 and GLT-1 labelled similar cellular compartments of the distal astrocytic processes and end feet, with a similar density and distribution in the human frontal cortex, and are a distinct and overlapping sub-population of astrocytes. Recent studies have suggested that AQP4 and GLT-1 exist in astrocytes as a macromolecular complex, concomitantly driving the accumulation of glutamate within astrocytes and protecting against neuronal excitotoxicity [45, 46]. Experiments of cultured astrocytes have also demonstrated that lowered concentrations or elimination of autoantibodies correlate with a down regulation of GLT-1 expression, suggesting a closely linked relationship between the two proteins [47].

Dysfunction of glutamate transporters in astrocytes leads to excessive glutamate release from neurons, which triggers neuronal death by excitotoxicity by the accumulation of excess glutamate. Astrocytes remove extracellular glutamate from the synaptic cleft through high-affinity excitatory amino acid transporters (1 and 2), which play a critical role in glutamate homeostasis and neuronal survival. Impairment of glutamate uptake by astrocytes leading to excitotoxicity has been linked to a number of neurodegenerative diseases, for example, AD, PD, HD [5, 31], and in temporal lobe epilepsy [32]. These studies suggest that abnormal protein aggregates inhibit glutamate uptake by astrocytes. In addition, decreased GLT-1 expression has been reported in preclinical models of AD, PD and HD, and reduced GLT-1 expression has been reported in the brains of AD and HD patients [32, 47, 48]. Similarly, a number of studies have reported altered AQP4-immunoreactivity in a range of disorders. For example, AQP4-immunoreactivity is increased in AD [47] and its mis-localisation is related to the formation of β-amyloid plaques [49], and reduced AQP4 expression has been reported in psychiatric and demyelinating disorders [5]. Recent studies have reported that increased AQP4 expression is a feature of the ageing brain and has associations with ARTAG pathology. Together, these studies suggest disruption of glutamate and water homeostasis contribute, at least in part, to the pathogenesis of a range of neurodegenerative diseases and brain ageing.

The current study has mapped the distribution and density of heterogeneous astrocytic populations in the frontal cortex with markers that label distinct cellular properties and cellular compartments of astrocytes, providing a reference map for future studies. Few studies have mapped the distribution of different astrocyte populations in the human brain and to date, most studies have focused on rodent and preclinical models. Further detailed anatomical mapping of diverse astrocyte populations across different cortical and subcortical regions will be informative to better understand their interactions with other cell types, regional regulation of homeostasis throughout the brain, how astrocytic dysfunction contributes to neurological diseases, what astrocyte subpopulations are primarily affected in disease and injury, which overlapping or distinct populations are vulnerable, what the common mechanisms involving astrocytes are across diseases, and what the best preclinical models are to probe these questions. Future studies may also focus on neurological diseases that preferentially involve astrocytes in disease-specific regions, for example, the tau protein abnormally accumulates in astrocytes and in different cellular compartments in the frontotemporal tauopathies [9]. Finally, a better understanding of astrocytes and their dysfunction in both health and disease would raise the possibility of different therapeutic options that might aim to target preserving astrocyte functions and their homeostatic support to neurons.

## Data Availability

The anonymised datasets generated during and/or analysed during the current study will be made available from the corresponding author on reasonable request from any qualified investigator.
